# Prevalence of venous thromboembolism in patients undergoing diagnostic venous ultrasound during the first SARS-CoV-2 pandemic: A personal history of VTE and a positive COVID-19 test are associated with the diagnosis of DVT

**DOI:** 10.1024/0301-1526/a001052

**Published:** 2023-01-20

**Authors:** Guglielmo LaTorre, Raveenjot Nagra, Haren Wijesinghe, Gowshan Rajeswaran, Jain Riya, Scerif Abdulkhaliq, Tom Barker, Arul Ganeshan, Robert Goudie, Nasr Hosaam, Alok Tiwari, Maciej Tadeusz Juszczak

**Affiliations:** 1 University Hospital Birmingham NHS Foundation Trust, Birmingham, UK; 2 Royal Centre for Defence Medicine, Queen Elizabeth Hospital, Birmingham, UK; 3 Medical Research Council Biostatistics Unit, Cambridge, UK; 4 Institute of inflammation and ageing University of Birmingham, UK

**Keywords:** SARS-CoV-2, COVID-19, venous thromboembolism, diagnostic imaging, prophylaxis, antiplatelet agents, Wells score

## Abstract

**Background:**

Venous thromboembolism appears to be associated with severe COVID-19 infection than in those without it. However, this varies considerably depending on the cohort studied. The aims of this single-centre, multi-site retrospective cross-sectional study were to assess the number of all venous scans performed in the first month of pandemic in a large university teaching hospital, to evaluate the incidence of deep venous thrombosis (DVT), and assess the predictive ability of the clinical information available on the electronic patient record in planning work-up for DVT and prioritising ultrasound scans.

**Patients and methods:**

All consecutive patients undergoing venous ultrasound for suspected acute DVT between 1st of March and 30th of April 2020 were considered. Primary outcome was the proportion of scans positive for DVT; the secondary outcomes included association of a positive SARS-CoV-2 PCR test, demographic, clinical factors, and Wells scores.

**Results:**

819 ultrasound scans were performed on 762 patients across the Trust in March and April 2020. This number was comparable to the corresponding pre-pandemic cohort from 2019. The overall prevalence of DVT in the studied cohort was 16.1% and was higher than before the pandemic (11.5%, p=.047). Clinical symptoms consistent with COVID-19, irrespective of the SARS-CoV-2 PCR test result (positive_COVID_PCR OR 4.97, 95%CI 2.31–010.62, p<.001; negative_COVID_PCR OR 1.97, 95%CI 1.12–3.39, p=.016), a history of AF (OR 0.20, 95%CI 0.03–0.73, p=.037), and personal history of venous thromboembolism (VTE) (OR 1.95, 95%CI 1.13–3.31, p=.014), were independently associated with the diagnosis of DVT on ultrasound scan. Wells score was not associated with the incidence of DVT.

**Conclusions:**

Amongst those referred for the DVT scan, SARS-CoV-2 PCR test was associated with an increased risk of VTE and should be taken into consideration when planning DVT work-up and prioritising diagnostic imaging. We postulate that the threshold for imaging should possibly be lower.

## Introduction

SARS-CoV-2 is a pan-vascular disease causing significant cardiovascular consequences [1, 2]. These include venous thromboembolism (VTE) in the deep venous system (deep vein thrombosis; DVT) or primary thrombosis in the lungs, pulmonary embolism (PE) and thrombosis in the venous microvascular systems [3]. Interestingly, no excess arterial events have been recorded [4].

The cardiovascular manifestations associated with SARS-CoV-2 infection are reflected at a biochemical level. Several studies have reported abnormal coagulation pro-files in patients with COVID-19 with elevated D-dimer, fibrinogen levels, decreased prothrombin time (PT) and abnormal activated partial thromboplastin time (aPTT) [5, 6].

The thrombotic complications appear to be highly prevalent amongst patients with severe COVID-19 infection. A meta-analysis of 42 studies representing 8721 patients documented prevalence of VTE amongst hospitalised patients to be 21%, with an overall DVT rate of 20% and the PE rate of 13% [7]. The high prevalence of DVT was explained by the higher detection rate associated with the use of compression ultrasound (CUS) as a screening tool in aiding the diagnosis. However, a 2020 guideline and expert panel report recommended that screening ultrasound should not be performed in patients with COVID-19, due to infection risks for those performing tests [8].

The aims of this study were to assess the number of all venous scans performed in the first months of pandemic in a large teaching Hospital Trust, to evaluate the incidence of DVT, and assess the predictive value of the clinical information available on the electronic patient record in prioritising the scans.

## Patients and methods

The manuscript was prepared according to STROBE guidelines for reporting of observational studies [9].

This study was conducted within the clinical audit framework (Birmingham Heartlands Hospital audit number 5190); no intervention was performed, and patients were not contacted outside their routine clinical care. Therefore, a specific ethical approval was not required, and patient consent was not sought in line with guidance from the UK Health Research Authority and UK Policy Framework for Health and Social Care Research.

### Study design and setting

This was a single centre, multi-site cross-sectional cohort study. We included all patients who underwent diagnostic imaging for suspected DVT between 1^st^ of March and 30^th^ of April 2020 in a large teaching Hospital Trust in the UK. This included upper and lower limb venous doppler ultrasound (DUS) and compression ultrasound. An additional cohort of similar size from 2019 was analysed to compare overall numbers and incidence of DVT.

### Study cohort

All patients who attended the radiology department for diagnostic imaging of acute DVT were identified in the departmental database (CRIS/PACS) based on pre-selected procedural codes.

To ensure that the selection bias did not affect our sample we compared it with the number of scans performed between 1 October and 31 December 2019. We also compared the prevalence of DVT between 2019 cohort with a subset of asymptomatic patients (not tested for COVID disease) from 2020.

### Data sources

Demographic data (age, sex, and ethnicity) were extracted from electronic health record (EHR: PICS and Dendrite).

Clinical data including smoking status, weight, height, Body Mass Index (BMI), cardiovascular and respiratory disorders, diabetes, presence of malignant disease, pregnancy, recent surgery within 3 months were extracted from EHR. Cardiovascular medications including antiplatelet agents, direct oral anticoagulants, warfarin, lipid-lowering agents such as statins, beta blockers, angiotensin receptor blocker agents and ACE-inhibitors were recorded. Hospital EHR, admission clerking documentation and scanned documentation were the primary source of data. The General Practice clinical record was cross-checked to ensure data completeness and accuracy when necessary.

Wells Score was obtained from radiology request forms and presenting symptoms recorded in the clinical documentation.

Admission status was defined as outpatient (scans requested through GP practices, outpatient clinics, and inpatients where the hospital stay was less than 24 h) and inpatient (all patients requiring hospitalisation for more than 24 h).

#### Identification of SARS-Cov-2 positive patients

Diagnosis of SARS-CoV-2 infection was based on the hospital electronic records confirming positive SARS-CoV-2 PCR test. Patients were stratified into three categories: 1) Symptomatic – tested negative, 2) Symptomatic – tested positive, 3) Asymptomatic – not tested.

### Primary outcome

The primary outcome was the proportion of new diagnoses of deep vein thrombosis out of all diagnostic imaging studies performed during the period of the study.

### Secondary outcomes

The secondary outcomes included association of a positive SARS-CoV-2 PCR test, demographic, clinical factors, and Wells score with the diagnosis of DVT.

### Statistical analysis

Results were analysed in pseudonymous format using R statistical environment [10] using pre-specified data analysis plan. Data characteristics were assessed using dplyr package [11].

Continuous variables were presented as median (interquartile range; IQR) unless indicated otherwise; categorical data were presented as frequencies (proportions; %). Student’s t-test and Wilcoxon rank-sum test were used to compare continuous data depending on Shapiro-Wilk normality test. Pearson’s chi-squared test and Fisher’s exact test with continuity correction were used to analyse categorical data.

Association of demographic and clinical factors with the diagnosis of DVT was examined using logistic regression. Multi-variate explanatory model was built using purposeful, manual selection of covariates with univariate p<0.1, taking into consideration the quality of the data and clinical judgement. Effect size was presented when appropriate as odds ratio (OR) with 95% confidence intervals (CI) and interpreted as “small” (OR<1.5), “medium” (1.5=<OR<5.0) and “large” (OR>=5.0).

Conditional inference trees [12] were used to select unbiased set of variables associated with the diagnosis of DVT.

## Results

### Data characteristics

**Figure 1:**
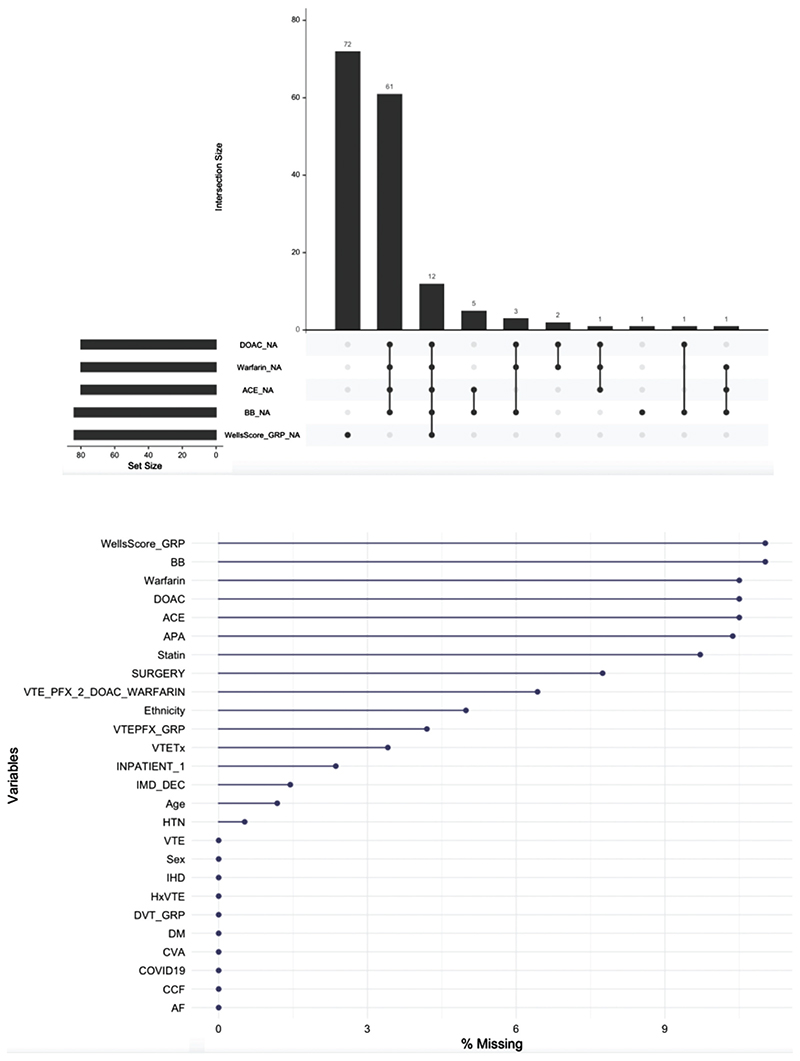
Data missingness. ACE: Angiotensin Converting Enzyme inhibitor; AF: atrial fibrillation; APA: antiplatelet agent; BB: beta-blocker; CCF: Congestive Cardiac Failure; COVID19: SARS-CoV-2 PCR testing status; CVA: Cerebrovascular Accident; DM: diabetes mellitus; DOAC: Direct Oral Anticoagulant; DVT: Deep Vein Thrombosis; HTN: hypertension; HxVTE: personal history of VTE; IHD: ischaemic heart disease; VTE: Venous Thromboembolism; VTEPFX_GRP: VTE prophylaxis; VTETx: treatment for VTE.

The final dataset included 762 observations of 26 demographic and clinical variables with 539 (68.0%) complete cases, and 4.0% overall data missingness. Data on primary outcome was complete in all cases (Figure 1).

### Cohort characteristics

We identified 762 patients who had 819 ultrasound scans for a suspected, acute DVT between 1^st^ of March and 30^th^ of April 2020. This was comparable to the number of scans performed between October and December 2019 (n= 530).

The median age was 67 (53, 78), and 362 patients (47.5%) were male. Data on ethnicity was missing in 38 patients (5.0%). White Caucasians constituted 82.9% of the cohort (600/724 patients), Asian patients – 11.7% (85/724), Black and Afro-Caribbean – 4.6% (33/724) and mixed race – 0.8% (6/724).

Four hundred and twenty patients (55.1%) were scanned on outpatient basis, whereas 324 patients (42.5%) had scans performed during inpatient stay. The admission status could not be determined in 18 cases (2.4%).

Index of multiple deprivations 2019 [13], based on the postcode was retrieved for 751 patients (98.6%). Patients from 20% most deprived households constituted 44.3% of the cohort, whereas only 9.7% of patients were from 20% least deprived areas [14, 15]. Data characteristics were summarised in Table I.

### SARS-CoV-2 status

One hundred and sixty-four patients were suspected for COVID-19 and had SARS-CoV-2 PCR test (164/762; 21.5%). Of these, 42 patients were found positive (25.6%), while 122 were confirmed to be negative (74.4%).

### Prevalence of DVT

During the study period DVT was detected in 121 out of 762 patients (15.9%). The prevalence was 35.7% (15/42; 95%CI 21.6–52.0) in patients who tested positive for SARS-CoV-2, 20.5% (25/122; 95%CI 13.7–28.7) for patients tested negative, and 13.9% (83/598; 95%CI 11.2–16.9) in patients who were not tested (no clinical suspicion of COVID-19).

The prevalence of DVT in the studied cohort during the peak of pandemic was higher than in the audited period of 2019 (61/530; 11.5%, 95%CI 8.9–14.5; OR 0.68, 95%CI 0.48–0.94, p=.019). However, the prevalence of DVT in patients with no clinical suspicion of COVID-19 was similar (OR 0.81, 95%CI 0.56–1.15, p=.234). While 762 patients were considered for this study, 819 scans were performed. Occasionally, where clinicians and/or vascular scientists had reasonable clinical suspicion of DVT one or more repeat scans were performed. Where one patient had multiple scans, once a patient was found to be positive, this was considered as one-only positive: all scans performed on that patient, were considered as a single positive.

### Factors associated with diagnosis of DVT

Age, sex, and ethnicity were not associated with an increased risk of DVT. However, patients from the 10% of most deprived households had higher prevalence of DVT than patients from all other deciles combined (OR 1.69, 95%CI 1.13–2.52, p=0.010).

Clinical presentation consistent with COVID-19 was associated with higher prevalence of DVT (PCR test positive OR 3.45, 95%CI 1.72–6.71, p=<.001; PCR test negative OR 1.60, 95%CI 0.96–2.6.0, p=.062) compared with patients with no suspicion of SARS-CoV-2 infection.

Interestingly, a history of atrial fibrillation, ischaemic heart disease, and hypertension were all associated with lower prevalence of DVT (AF – OR 0.21, 95%CI 0.03–0.70, p=.009; IHD – OR 0.32, 95%CI 0.10–0.70, p=.004; HTN – OR 0.58, 95%CI 0.37-0.88, p=.014).

A personal history of VTE was significantly associated with higher prevalence of DVT (OR 2.19, 95%CI 1.35–3.49, p=.002).

Regular antiplatelet agents (irrespective of type), statins and ACEi/ARBs were associated with a lower incidence of DVT in our sample (APA – OR 0.25, 95%CI 0.10–0.51, p<.001; statin – OR 0.42, 95%CI 0.25-0.68, p<.001; ACEi/ARB – OR 0.42, 95%CI 0.23–0.71, p=.001). Direct oral anticoagulants and warfarin were not associated with the incidence of DVT.

**Figure 2:**
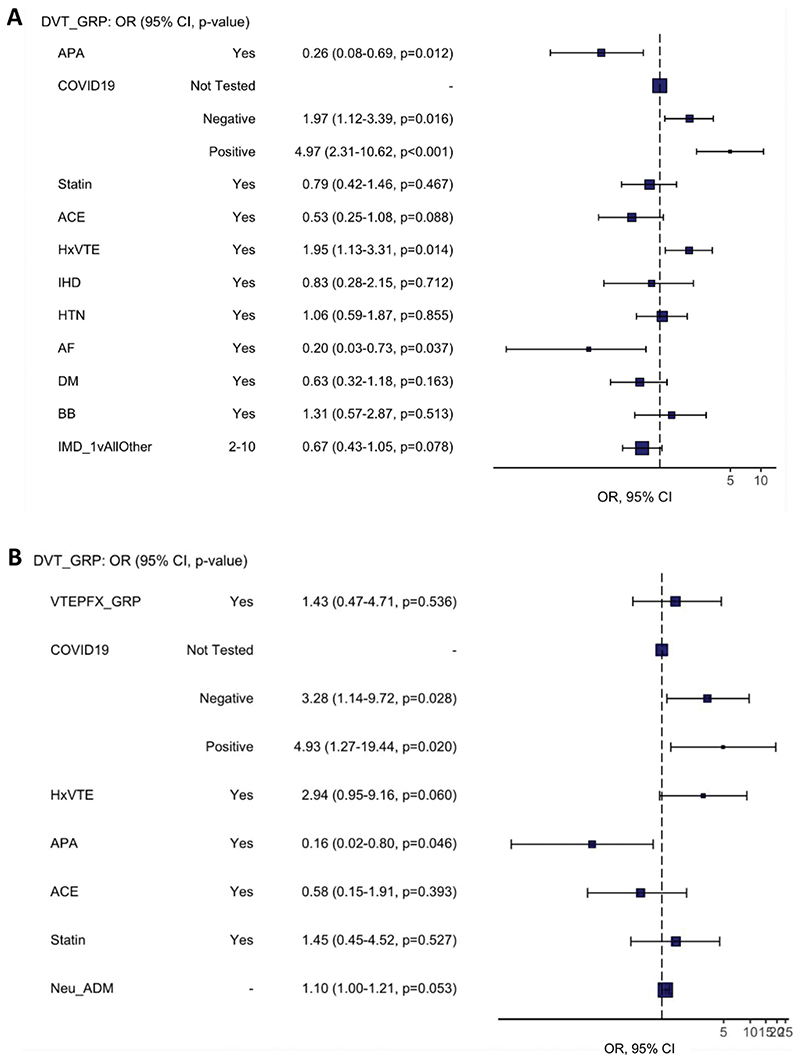
Forest plot visualising results of multivariable logistic regression in all patients (A) and inpatients (B). ACE: Angiotensin Converting Enzyme inhibitor; AF: atrial fibrillation; APA: antiplatelet agent; BB: beta blocking agent; COVID19: SARS-CoV-2 PCR testing status; DM: diabetes mellitus; HxVTE: personal history of VTE; HTN: hypertension; IHD: ischaemic heart disease; IMD_1vAllOther: Index of Multiple Deprivations 2019 (1st decile v. 2nd to 10th decile); OR: odds ratio; 95% CI: confidence interval.

Multivariable logistic regression, summarised in Table II, demonstrated that presence of symptoms necessitating PCR test (positive SARS-CoV-2 PCR OR 4.97, 95%CI 2.31–10.62, p<.001; negative SARS-CoV-2 PCR test OR 1.97, 95%CI 1.12–3.39, p=.016), personal history of VTE (OR 1.95, 95%CI 1.13–3.31, p=.014), were independently associated with the diagnosis of DVT on ultrasound scan (Figure 2). Concurrently, however, a history of AF (OR 0.20, 95%CI 0.03–0.783, p=0.037) and use of antiplatelet medications (OR 0.26, 95%CI 0.08–0.69, p=0.012) were associated with a decreased risk of DVT (Figure 2).

### VTE prophylaxis

The data on VTE prophylaxis was missing in 27 patients (8.3%) out of 324 inpatients. VTE prophylaxis (or anticoagulation – if patients were on it prior to admission) was prescribed for 185 out of 312 eligible patients (59.3%). There were no new diagnoses of DVT amongst patients who were not on VTE prophylaxis (or anticoagulation).

### Well’s score

Data on Wells score was missing in 84 out of 762 patients: 26 inpatients (8.0%), 56 outpatients (13.3%) and 2 with missing admission status (11.1%).

Amongst patients with DVT who were positive for SARS-CoV-2 the risk of DVT determined by Wells score was high in 46% (7/15) and intermediate in 53% (8/15). There were no patients with low risk in this group.

A majority of patients who tested negative for SARS-CoV-2 and developed DVT were categorised as high risk (66.7%; 16/24), with 33.3% in the intermediate risk group (8/24). No patients in this group had low risk of DVT. Details of Wells score was missing in one patient (4.0%).

Similarly, a majority of patients with DVT, who were not tested for SARS-CoV-2 due to absence of symptoms suggestive of COVID-19 were in the high-risk group (67.9%; 53/78) with only 25.6% (20/78), and 6.4% in low-risk group (5/78). The data on Wells score was missing in 5 patients (6.0%). The differences were not statistically significant.

In patients who did not develop DVT but were tested positive for SARS-CoV-2 the vast majority of patients were deemed high-risk (74.1%; 20/27) with 25.9% (7/27) in the intermediate risk group.

### D-Dimer levels

Two hundred and fifty-five patients (255/762; 33.5%) had D-Dimer test as part of their assessment. Sixteen results were below lower limit of quantification (LLOQ; 6.3%). Since this proportion was less than 10% in these cases we imputed the value LLOQ/2 [16].

A similar proportion of patients with (44/121; 36.4%) and without the diagnosis of DVT (211/641, 32.9%) had the D-Dimer test performed. The D-Dimer level was significantly higher in patients with DVT than in those with no DVT (2,455.5 [736.2, 5,162.0] v. 434 [271.5, 703.0], p<.001).

A similar proportion of D-Dimer tests were performed for patients with no COVID symptoms (206/598; 34.4%), as for patients with negative (39/122; 32.0% OR 1.11, 95%CI 0.74–1.71, p=0.675) and positive SARS-CoV-2 PCR test results (10/42; 23.8%; OR 1.66, 95%CI 0.83–3.65, p=0.179).

The D-Dimer levels were highest in patients with a negative PCR test, followed by those with the positive PCR test and the patients with no suspicion of COVID-19 (866 [445.0, 2,780.5] v. 688.5 [391, 1,585] v. 456.5 [276.5, 802.0], p<.001).

During our study period it was already understood that SARS-CoV-2 infection was associated with increased risk of VTE. As such, systematic, indiscriminate, D-Dimer testing outside of clinical trials was not carried out.

## Discussion

During first two months of the pandemic there were 819 compression ultrasound scans performed on 762 patients across the Trust. The overall prevalence of DVT in this cohort was 16% and was higher than before the pandemic (11.5%). We demonstrated that the referral pattern was similar to the one before the pandemic (both number of scans and DVT detection rate). Clinical symptoms consistent with COVID-19, irrespective of the SARS-CoV-2 PCR test result, were associated with a significantly higher prevalence of DVT. Regular antiplatelet agent prior to onset of symptoms prompting the scan was associated with lower prevalence of DVT. Wells score was not helpful in prioritising the scans, as it was not associated with the incidence of DVT. Very few calf DVTs were reported as calf veins were not routinely included in the compression scans as recommended by the National Institute of Clinical Excellence (NICE). However, if the scan was performed by specialised vascular scientists, in the vascular laboratory, deep calf veins might be scanned to provide comprehensive diagnostic information.

The increased incidence of VTE in patients with SARS-CoV-2 infection has been demonstrated in many reports [7, 17, 18]. The incidence was higher in critically ill patients than in patients not requiring higher level of care, or patients not requiring hospitalisation. The post-discharge incidence of VTE was also low, but the baseline incidence of VTE in the studied ethnic group is generally low [19]. With this in mind, there is a need to better understand the viral coagulopathy, with a focus on increased image directed detection of asymptomatic cases, adequate prevention, and treatment.

Our study was intentionally limited only to patients who underwent diagnostic imaging for acute DVT. We assumed that the clinical judgement of clinicians referring patients for diagnostic imaging has not changed much despite the pandemic. The only patients that clinicians would find it difficult to obtain scans for DVT are the critically ill patients on ITU and these were studied separately. We demonstrated a significant association of VTE with COVID-19 (clinical presentation rather than PCR test). This implies that a combination of any suspicion of DVT, with clinical picture suggesting COVID-19 should prompt clinicians to prioritise diagnostic imaging and instigate treatment.

A recent guideline and expert panel report recommended that screening ultrasound should not be performed in patients with COVID-19, especially those critically ill, due to the infection risk for those performing the tests [8]. We support this; however, as demonstrated in this study, the classical risk factors do not help prioritising scans. Thromboembolism seems to be an integral part of the COVID-19 clinical picture and indeed paucisymp-tomatic events can already be detected on presentation [20]. We believe that appropriate training should be offered to non-radiology trained clinicians to gain competencies in bedside compression ultrasound. This could reduce the burden of scans for diagnostic departments and facilitate early diagnosis and treatment of DVT without increasing the risk of disease transmission.

Various mechanisms of thrombo-embolism in patients with SARS-CoV-2 infection have been suggested [2]. Some proposed alterations in coagulation profiles and underlying genetic problems. The latter would be consistent with our findings showing a significant association of the diagnosis of DVT with the personal history of VTE. However, systemic hypercoagulation is not novel, and not exclusive to the SARS-CoV-2. Viral coagulopathy has been noted in other systemic viral infections such as the classical SARS-CoV-1 and 2009 H1N1, both specifically causing intrapul-monary thrombi [21, 22]. Hypercoagulability typically occurs as the result of increased coagulation or imbalance between coagulation and fibrinolysis. However, on its own, it may not result in thrombosis. Other elements of Virchow’s triad, including vessel wall damage and circulatory stasis play their part too.

Damaged vascular endothelium is a known activator of the coagulation cascade. SARS-CoV-2 is able to bind to the angiotensin-converting enzyme-2 (ACE-2) receptor expressed on a variety of cell types including alveolar epithelial cells, cardiomyocytes, and vascular endothelial cells, causing their activation. Viral replication causes inflammatory activation of the endothelial cell. Interestingly, the SARS-CoV-2 replication increases expression of heparanase and cleavage of heparan sulphate from the cell surface [23]. It is likely that like other viruses, SARS-CoV-2 gets trapped by heparan sulphate and requires heparanase for cleavage and a full release. Heparan sulphate is a biologically diverse glycosaminoglycan abundantly present on the surface of mammalian cells and takes part in multiple cellular processes. It is not heparin but heparan sulphate that modulates anticoagulation in the intravascular compartment. Heparin is present mainly in the secretory granules of basophils and mast cells and is secreted following injury. Cleavage of heparan sulphate from endothelial cell surface disrupts multiple pathways including cell signalling, adhesion and modulation of thrombosis/coagula- tion. This reduces anticoagulant, anti-thrombotic and anti-adhesive properties of the endothelium allowing leukocytes and platelets to aggregate on its surface initiating thrombus formation [24]. This could lead to adhesion of leukocytes to endothelium and aggregation of platelets in low-flow areas, such as small venules resulting in pau-cisymptomatic events. Increased viral replication occurring with progression of the disease, can increase the area of damaged endothelium and allow thrombosis to propagate. This in turn, can result in symptomatic events. It is plausible that the association of antiplatelet agents with lower prevalence of thrombosis seen in our study relates to prevention of the platelet aggregation in the asymptomatic and paucisymptomatic phase of the disease and prevents propagation of thrombosis to large VTE.

Interestingly, our results contradict those published by Sahai *et al*. who demonstrated a prothrombotic effect of aspirin [25]. In their analysis they combined those who were on aspirin prior to contact, with those who were recently started on it, without considering indications. The direction of the association could significantly confuse interpretation of the results. For instance, they made the observation that antiplatelet therapy was associated with a higher incidence of stroke. This is certainly true; however, the timing plays a very important role in this relationship. All patients who develop stroke and who have no specific contraindications are started on high-dose antiplatelet regimen. If the timing of these two events is not known, it is easy to conclude that aspirin caused the stroke. We believe this is not the case. In our study, we only recorded use of aspirin prior to contact to avoid such problems. Utilisation of large, highly granular chronological datasets based on routinely collected data (DECOVID/PIONEER) may help to explain the observed phenomena and assess anticoagulant and antithrombotic strategies.

The more recent RECOVERY trial [26] needs to also be considered. The first of its kind (randomised, controlled and open-label), the RECOVERY trial set to investigate treatments for COVID. In the aspirin arm of the trial, patients were randomised to wither standard treatment or standard treatment plus 150 mg aspirin, orally or rectally, once a day. The trial was analysed as intention to treat. 7351 patients received aspirin (vs 7541 who received standard care). Aspirin treatment did not affect overall mortality rates or mortality, however the authors did record an absolute reduction in thrombotic events of 0.6%. Our results are difficult to compare to this major trial as primary endpoints did not include venous thromboembolism.

We believe that as we phase out of the global pandemic, the testing for SARS-CoV-2 is also going to phase out. This data provides an important insight into the management of patients who are symptomatic, but do not have the immunological/molecular evidence of infection.

## Limitations

We recognise that our study has significant limitations. This was a retrospective audit, and all data relied on accurate recording of clinical details. We validated the cases where there was a suspicion of inaccuracy and crosschecked the information with community databases whenever possible.

Secondly, the dataset is small, limited by the observation period and the selection of cohort of interest. This study does not account for the severity of SARS-CoV-2 infection, duration of thromboprophylaxis or therapeutic anticoagulation which could potentially reduce the thromboembolic events. The dataset does not have necessary granularity to make such observations.

Moreover, we were unable to retrieve the data for the pre-pandemic cohort with regards to numbers of inpatient and outpatient scans. We are therefore unable to state confidently that this ratio of inpatient and outpatient scans is comparable between the two cohorts.

Furthermore, during the time of study, diagnostic criteria for COVID were still being developed. Therefore, we cannot comment on the proportion of both false negatives and false positives.

Finally, we also recognise that the Well’s score has limited use in the inpatient setting as it was not found to be as strongly predictive as in the outpatient setting [27].

## Conclusions

Symptoms consistent with COVID-19 irrespective of SARS-CoV-2 PCR result, but not the clinical risk assessment (Wells score) are associated with an increased risk of VTE and should be taken into consideration when planning DVT work-up and prioritising diagnostic imaging.

APA may play a role in the prevention of COVID-19- associated venous thromboembolic events.

We postulate that appropriate training of medical staff in performing bedside compression ultrasound scans (CUS) may help increase DVT detection rates. This in turn should help instigating early treatment of VTE without increasing the risk of SARS-CoV-2 transmission and help managing the workload within diagnostic radiology.

## Figures and Tables

**Table I:** Cohort characteristics

Variable	Level	Median [IQR]/mean±SD/proportion (%)
Age		67 [53, 78]
Sex	Female	400 (52.5)
	Male	362 (47.5)
Ethnicity	White	600 (82.9)
	Asian	85 (11.7)
	Black	33 (4.6)
	Mixed	6 (0.8)
Comorbid status	Ischaemic heart disease	95 (12.5)
	Atrial fibrillation	51 (6.7)
	Congestive cardiac failure	41 (5.4)
	Cerebrovascular accident	58 (7.6)
	Hypertension	284 (37.5)
	Diabetes	160 (21.0)
	Personal history of VTE	112 (14.7)
Medications	Antiplatelet agent	128 (18.7)
	Direct anticoagulant (DOAC)	65 (9.5)
	Warfarin	16 (2.3)
	Statin	238 (34.6)
	ACEi/ARB	178 (26.1)
	Beta blocker	102 (15.0)
Wells Score	Low	5 (4.3)
	Intermediate	211 (31.1)
	High	428 (63.1)
D-Dimer* Deep Vein Thrombosis		523 [284, 967]
	Upper Limb	15 (12.4%)
	Ilio-femoral	45 (37.2%)
	Femoro-Popliteal	47 (38.8%)
	Infrapopliteal	14 (11.6%)

*Notes*. ACEi: angiotensin converting enzyme inhibitor; aPTT: activated partial thromboplastin time; ARB: angiotensin receptor blocker; IQR: interquartile range; PT: prothrombin time; SD: standard deviation; VTE: venous thrombo-embolism.

**Table II:** Univariable and multivariable analysis of factors associated with the diagnosis of DVT on ultrasound scan

Variable	Level	Univariable p	Multivariable analysis	
			OR (95%CI)	p-value
Age		0.195		
Sex	Female	0.318		
	Male			
Ethnicity	White	0.477		
	Asian			
	Black			
	Mixed			
IMD COVID-19 status	Dec1 v Dec2-10		0.67 [0.43;1.05]	0.078
	Not suspected	<0.001	Ref	
	Negative		1.97 [1.14;3.41]	0.016
	Positive		4.97 [2.33;10.61]	<0.001
Comorbid status	IHD	0.008	0.83 [0.30;2.26]	0.712
	AF	0.024	0.20 [0.04;0.91]	0.037
	CCF	0.174		
	CVA	0.489		
	HTN	0.015	1.06 [0.59;1.88]	0.855
	DM	0.077	0.63 [0.33;1.20]	0.163
	VTE	0.001	1.95 [1.14;3.33]	0.014
Medications	APA	<0.001	0.26 [0.09;0.75]	0.012
	DOAC	0.633		
	Warfarin	0.906		
	Statin	<0.001	0.79 [0.42;1.48]	0.467
	ACEi/ARB	0.002	0.53 [0.25;1.10]	0.088
	BB	0.084	1.31 [0.58;2.93]	0.513
Wells’score	Low	0.734		
	Intermediate			
	High			
D-Dimer*		<0.001		

*Notes*. *D-Dimer, PT and neutrophil count were omitted from multivariable analysis of all patients due to high data missingness. ACEi: angiotensin converting enzyme inhibitor; AF: atrial fibrillation; APA: antiplatelet agent; aPTT: activated partial thromboplastin time; ARB: angiotensin receptor blocker; BB: beta blocking agent; CCF: congestive Cardiac Failure; CVA: cerebrovascular accident (stroke/transient ischaemic attack); DM: diabetes mellitus; DOAC: direct anticoagulant; HTN: hypertension; IMD: index of multiple deprivations 2019; IQR: interquartile range; PT: prothrombin time; SD: standard deviation; VTE: venous thromboembolism.
